# Identifying existing approaches used to evaluate the sustainability of evidence-based interventions in healthcare: an integrative review

**DOI:** 10.1186/s13643-022-02093-1

**Published:** 2022-10-15

**Authors:** Rachel Flynn, Bonnie Stevens, Arjun Bains, Megan Kennedy, Shannon D. Scott

**Affiliations:** 1 Faculty of Nursing, Level 3, Edmonton Clinic Health Academy, University of Alberta, 11405 87 Avenue, Edmonton, Alberta T6G 1C9 Canada; 2 Lawrence S Bloomberg Faculty of Nursing and Faculties of Medicine and Dentistry, University of Toronto, Toronto, Canada; 3 Associate Chief Nursing Research & Senior Scientist, Research Institute, The Hospital for Sick Children, 686 Bay St., Toronto, ON M5G 0A4 Canada; 4 John W. Scott Health Sciences Library, 2K312 WMC University of Alberta, Edmonton, AB T6G 2R7 Canada

## Abstract

**Background:**

There is limited evidence to evaluate the sustainability of evidence-based interventions (EBIs) for healthcare improvement. Through an integrative review, we aimed to identify approaches to evaluate the sustainability of evidence-based interventions (EBIs) and sustainability outcomes.

**Methods:**

Following Whittemore and Knafl’s methodological process: (1) problem identification; (2) literature search; (3) data evaluation; (4) data analysis; and (5) presentation, a comprehensive search strategy was applied across five databases. Included studies were not restricted by research design; and had to evaluate the sustainability of an EBI in a healthcare context. We assessed the methodological quality of studies using the Mixed Methods Appraisal Tool.

**Results:**

Of 18,783 articles retrieved, 64 fit the inclusion criteria. Qualitative designs were most commonly used for evaluation (48%), with individual interviews as the predominant data collection method. Timing of data collection varied widely with post-intervention data collection most frequent (89%). Of the 64 studies, 44% used a framework, 26% used a model, 11% used a tool, 5% used an instrument, and 14% used theory as their primary approach to evaluate sustainability. Most studies (77%) did not measure sustainability outcomes, rather these studies focused on sustainability determinants.

**Discussion:**

It is unclear which approach/approaches are most effective for evaluating sustainability and what measures and outcomes are most commonly used. There is a disconnect between evaluating the factors that may shape sustainability and the outcomes approaches employed to measure sustainability. Our review offers methodological recommendations for sustainability evaluation research and highlights the importance in understanding mechanisms of sustainability to advance the field.

**Supplementary Information:**

The online version contains supplementary material available at 10.1186/s13643-022-02093-1.

## Background

The translation of evidence-based interventions (EBIs) to healthcare practices takes an average of 17 years [1]. As a result, the field of implementation science (IS) seeks to understand ways to improve the implementation and dissemination of EBIs in healthcare [2]. As IS has matured, researchers have recognized that implementation, which often requires substantial resources, is meaningless without long-term sustainability efforts [3]. We draw upon a comprehensive definition for sustainability by Moore et al. [4] who define sustainability under five constructs: (1) as occurring after a defined period of time, (2) where the intervention, and/or implementation strategies continue to be delivered, (3) behavior change is maintained, (4) the program and behavior change may evolve or adapt while (5) continuing to produce benefits for individuals/systems. Policy-makers and other stakeholders are increasingly concerned with the long-term impact of such investments in EBIs [5]. Sustainability is a key outcome of the implementation process [5], and a priority topic for IS. Yet, our understanding of how to evaluate the sustainability of an EBI in healthcare remains limited in implementation research.

Recent synthesis efforts have focused on (a) identifying sustainability approaches (i.e. theory, model, framework), (b) how sustainability approaches have been used [6, 7], (c) identifying sustainability determinants [6–9], or sustainability strategies [10] or (d) methods of sustainability measurement [11]. Despite these recent efforts, there is not a comprehensive synthesized approach on how to evaluate sustainability of EBIs. This work is critical to inform sustainability planning, implementation, and evaluation of EBIs in healthcare.

### Aim

The aim of this integrative review was to identify and synthesize approaches that have been used to evaluate the sustainability of EBIs in healthcare. We aimed to enhance our understanding of sustainability definitions, research designs, data collection methods, timing, measures, and sustainability outcomes used for sustainability evaluation studies.

### Design

We conducted an integrative review that followed Whittemore and Knafl’s [12] five-stage methodological process: (1) problem identification; (2) literature search; (3) data evaluation; (4) data analysis (data reduction, display, comparison, and conclusions); and (5) presentation. Integrative reviews can include diverse data sources and do not restrict the type of study design. Furthermore, integrative reviews take a more flexible approach to analysis compared to systematic reviews, combining both quantitative and qualitative data if there are similarities [12–14].

## Methods

### Literature search

We conducted a systematic database search using comprehensive strategies, including diverse data sources and methodologies, to advance the understanding of sustainability evaluation as it relates to health EBIs. In December 2018 and July 2020, we searched the following databases: Ovid MEDLINE(R) and Epub Ahead of Print, In-Process and Other Non-Indexed Citations and Daily (1946 to current); OVID Embase (1974 to current); EBSCOhost CINAHL Plus with Full-text (1937 to current); Wiley Cochrane Library (inception to current). A health research librarian conducted the search in consultation with the research team. We combined terms representing sustainability with terms representing knowledge translation or knowledge implementation of healthcare programs and terms related to evaluation or measurement. An agreed-upon set of terms allowed us to exclude as many irrelevant studies as possible without eliminating relevant ones. For example, the terms excluded studies representing environmental sustainability, patient institutionalization, and animal studies from the primary set of results. Results were limited to the English language and to academic journals (when the interface permitted). We also used a snowball approach to manually search reference lists of relevant systematic reviews to identify additional relevant sustainability evaluation studies.

The initial database search in December 2018 generated a total of 13,613 records. We identified 5399 duplicate records from this batch, leaving 8214 records for title/abstract screening. An update of the search was performed in July 2020 using the same original databases and search strategies. We found 5170 new items from the updated search and removed a further 2718 duplicate records, leaving 2452 items remaining. Full search details can be found in Additional file 1. See Additional file 2 for the completed PRISMA checklist.

### Inclusion and exclusion criteria

We applied the inclusion and exclusion criteria (Table 1) during screening. We included studies that focused on implementation, dissemination, impact, uptake, scale and spread, testing and monitoring; but, studies had to have an independent sustainability evaluation component.

**Table 1:** Inclusion and exclusion criteria

Inclusion criteria	Exclusion criteria
Articles were included if they: • Were published in a peer-review journal • Were primary research • Had explicit research design and data collection methods • Had an explicit theoretical approach^a^ for sustainability (theory^b^, model^c^, framework^d^, instrument^e^, method, checklist, process, strategy, conceptualizations, development, pilot test and/or tool^f^) • Were within a healthcare setting^g^ • Evaluated the sustainability of an EBI (Note that inconsistent terminology for EBI is prominent and thus may be referred to as a QI intervention/ initiative in the literature).	Articles were excluded if they: • Were secondary research • Were gray literature • Were outside a healthcare context (e.g., schools, social care settings) • Did not evaluate the sustainability of an EBI or QI intervention using a defined approach or, • Did not evaluate sustainability using a clear research design and method • Did not have an independent evaluation component on sustainability • Focused only on the sustainability of clinical outcome without a tangible approach • Focused only on defining or constructing concepts of sustainability

^a^Defined as a process for describing and/or guiding the process of translating research into practice (process models); understanding and/or explaining what influences implementation outcomes (determinant frameworks, classic theories, implementation theories); and evaluating implementation (evaluation frameworks) [14]

^b^Defined as a theoretical approach in implementation science with some predictive capacity (e.g., to what extent … ?) and attempts to provide an enhanced understanding and explanation of certain aspects of implementation [14]

^c^Defined as a theoretical approach in implementation science commonly used to describe and/or guide the specific, step-by-step, process of translating research into practice [14]

^d^Defined as a theoretical approach in implementation science with a descriptive purpose [14]. Points out factors believed or found to influence implementation outcomes but does not specify the mechanisms of change [14]

^e^Facilitates evaluation and usually evolves as an extension of frameworks, or models, or to operationalize theories [14]

^f^Assists individuals on how to retrieve, comprehend, and implement research evidence [14]

^g^Defined as a broad array of services and places where healthcare provision occurs, including acute care hospitals, urgent care centers, rehabilitation centers, nursing homes and other long-term care facilities, specialized outpatient services (e.g., hemodialysis, dentistry, podiatry, chemotherapy, endoscopy, and pain management clinics), and outpatient surgery centers

### Data extraction, analysis, and synthesis

We used Endnote X7 as the management system for this review. After removing duplicates, we conducted a two-stage screening process of the citations retrieved from our database searches. In the first screening stage, one reviewer (RF) independently screened the abstracts and titles of all the citations retrieved from the database searches. A second reviewer (AB) independently screened a randomly selected 10% of all titles and abstracts to verify selection for inclusion or exclusion. In the second stage, two reviewers (RF and AB) independently screened all full-text articles that had passed first-stage screening. We discussed any differences in screening at team meetings and refined our inclusion and exclusion criteria to reflect these discussions.

The two reviewers independently extracted the following variables: (1) study design, (2) evaluation type (independent versus composite), (3) sustainability definition and terms used, (4) type and name of theoretical approach, (5) purpose of approach use, (6) data collection methods, (7) timing of evaluation data collection (e.g., pre-, and/or post-implementation of intervention), (8) reported sustainability measures and, 9. reported sustainability outcomes.

### Theoretical approach used to evaluate sustainability

During extraction, we applied Nilsen’s five categories of theoretical approaches [14] used in implementation science (Table 2) to extract the primary theoretical approach that studies used to evaluate sustainability. Furthermore, we have also included tools and instruments as additionally accepted approaches to sustainability evaluation. It should be noted that Nilsen uses the term theoretical approach as a broad concept, which includes theories as one of many approaches. We will use the term ‘theoretical approach’ to describe all approaches to sustainability evaluation, including models, theories, frameworks, tools and instruments. If the primary theoretical approach was not explicitly stated by the author to be based on a theory, model, or framework, or multiple theoretical approaches were used, the most focused-on theoretical approach was deemed primary. We also extracted measures used to evaluate sustainability as reported by the authors of the included studies.

**Table 2:** Categories of theoretical approaches used in implementation science

Theoretical approach [14]	Definition [14]
Process Models	Specify steps in the process of translating research into practice [14].
Determinant frameworks	Classes or domains of determinants that are hypothesized or have been found to influence implementation outcomes [15].
Classic theories	Describe how change occurs without ambitions to carry out the change [14].
Implementation theories	Developed and adapted by researchers for potential use in implementation science to achieve enhanced understanding and explanation of certain aspects of implementation [14].
Evaluation frameworks	Provide a structure for evaluating implementation endeavors [14].

### Sustainability measures and outcomes

We extracted whether an included study evaluated (a) sustainability determinants and (b) sustainability outcomes and how they measured these variables. We defined sustainability determinants as correlates and predictors of sustainability (organizational, contextual, and strategies) and sustainability outcomes as the subsequent impact (healthcare improvement or public health outcomes) of sustained intervention use [5]. To further unpack sustainability outcomes, we extracted and synthesized nine sustainability outcomes across the 64 included studies (Table 3) [6, 15, 16].

**Table 3:** Sustainability outcomes identified in included studies [6]

1. Benefits for patients, staff, and stakeholders continue.	
2. Initiative activities or components of the intervention continue.	
3. Maintenance of relationships, partnerships, or networks.	
4. Maintenance of new procedures, and policies.	
5. Attention and awareness of the problem or issue is continued or increased.	
6. Replication, roll-out, or scale-up of the initiative.	
7. Capacity built within staff, stakeholders, and communities continues.	
8. Adaptation in response to new evidence or contextual influences.	
9. Gaining further funds to continue the initiative and maintain improvements.	

Data analysis followed the methodological steps outlined by Whittemore and Knafl [12] which included data reduction, data display, data comparison, and drawing conclusions and verifications. We compared, grouped, and synthesized each study by these variables. Evidence tables were created to summarize and describe the studies included in this review.

### Quality appraisal

We used the Mixed Methods Appraisal Tool (MMAT) [17] to assess the methodological quality of studies. Two reviewers independently completed MMAT assessments and compared scoring. The MMAT [17] appraises the quality by study design type, such as quantitative, qualitative, or mixed empirical methods. We distinguished mixed-methods as studies combining both qualitative and quantitative methods, whereas studies classified as multi-methods used 2+ qualitative methods. The criteria are specific to each type of study, with five domains apportioned to qualitative studies and quantitative studies subdivided into randomized controlled, non-randomized and descriptive studies. Each study is assigned an overall quality score, using asterisks representing the quality appraisal of each study. Scores vary from 20% (*) when one criterion is met to 100% (*****) when all criteria are met. Studies were not excluded based upon MMAT [17] ratings/scores. The purpose of conducting the MMAT appraisals was to get a sense of the quality of the research on this topic.

## Results

**Fig. 1:**
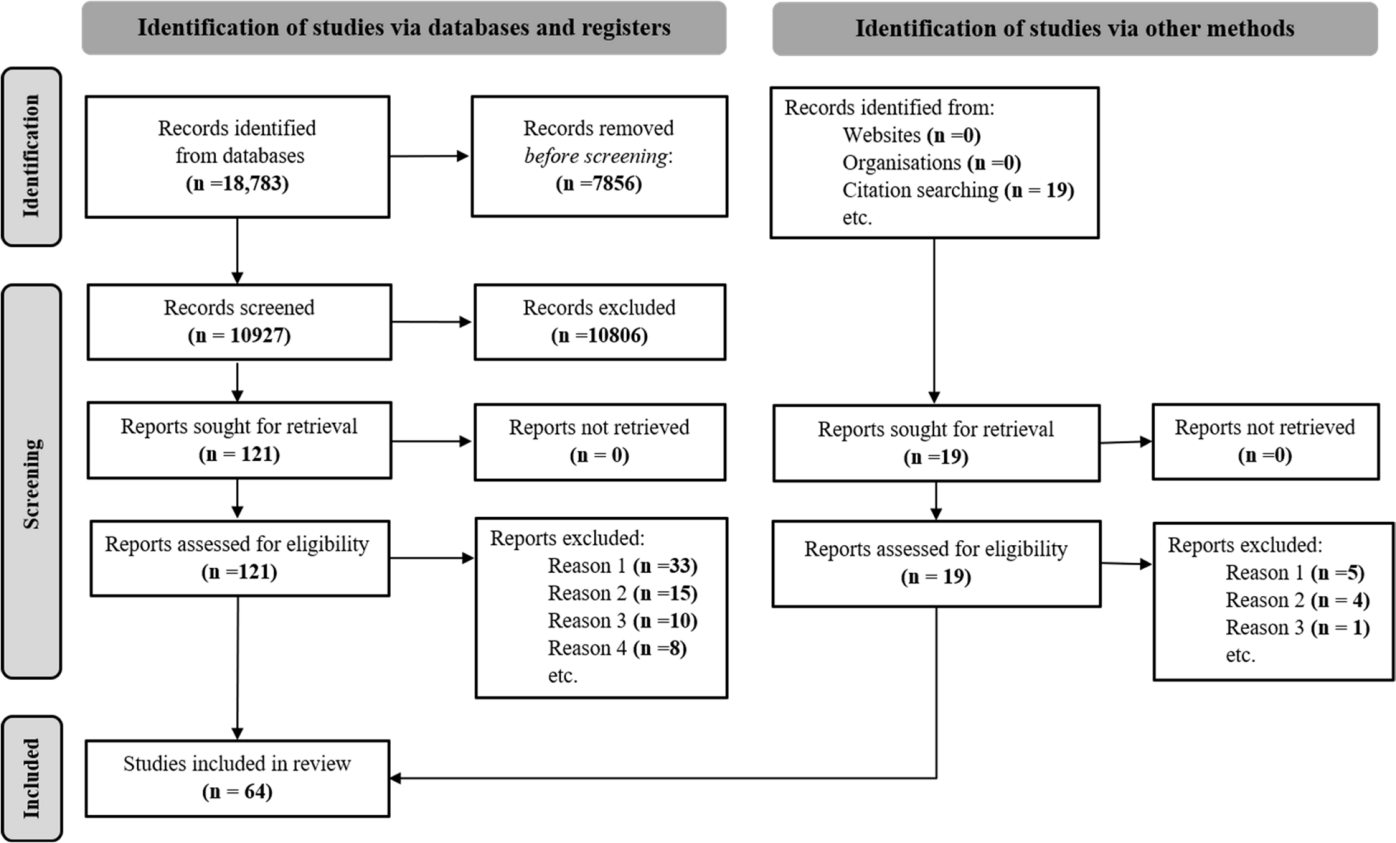
PRISMA 2020 flow diagram of search results

Of the total 18,783 records identified through database searching, 64 studies were included for our review. Figure 1 depicts our search, screening, and selection results using the Preferred Reporting Items for Systematic Reviews and Meta-analyses (PRISMA) guidelines flow diagram [18]. Our results are presented under five headings according to our research aims. Full citation list for the 64 studies can be found in Additional file 3.

### Data collection methods

Of the included studies, 49% (*n* = 31) were qualitative, making this the most frequently used research design [19–49]. One quarter (25%; *n* = 16) used a mixed-methods design [50–65], followed by 14% (*n* = 9) studies that used a multi-methods design [66–74]. Only 12% (*n* = 8) of the included studies used a quantitative research design [75–82]. Of the 31 qualitative studies, 94% (*n* = 29) used interviews to collect evaluation data [19–22, 24–40, 42–49]; however, only 59% (*n* = 17) of those reported interviews as the sole data collection method [20, 21, 24–27, 29, 30, 32, 33, 37, 38, 40, 43, 45, 47, 48]. The remaining 3 qualitative studies used an onsite inspection and assessment tool [41], steering-committee meeting minutes [23], and a combination of workshop sessions and field notes [49]. Of the 16 mixed methods studies [50–65], 88% (*n* = 14) used interviews, although never as the only method of data collection [50–52, 55–65]. Interviews were accompanied by surveys or questionnaires in 79% (*n* = 11) of the 14 studies using interviews [50–52, 55, 58–62, 64, 65]. Interviews were also the most common method of data collection in the nine multi-methods studies [66–74], with 66% (*n* = 6) combining interviews with another method [67–70, 72, 73]. Surveys or questionnaires accompanied the interviews in 22% (*n* = 2) of studies [69, 73]. Of the nine quantitative studies, 100% (*n* = 8) administered a survey or questionnaire to collect sustainability evaluation data [75–82] Table 4

**Table 4:** Data collection method of included studies

Data collection method		Referenced studies
**Qualitative**	**49% (*n*** **= 31)**	19–49
Interviews	*n* = 29	19–22, 24–40, 42–49
Onsite inspection + tool	*n* = 1	41
Steering committee minutes	*n* = 1	23
Workshops + field notes	*n* = 1	49
**Mixed methods**	**25% (*n*** **= 16)**	50–65
Interviews + survey/questionnaire	*n* = 11	50–52, 55, 58–62, 64, 65
**Multi methods**	**14% (*n*** **= 9)**	66–74
Interviews + survey/questionnaire.	*n* = 2	69, 73
Interviews + other	*n* = 6	67–70, 72, 73
**Quantitative**	**12% (*n*** **= 8)**	75–82
Survey/questionnaire	*n* = 8	75–82

### Sustainability definitions and terms in evaluation studies

Of the 64 included evaluation studies, 61% (*n* = 39) provided a clear definition for sustainability [19–21, 27–36, 39, 42–50, 53, 54, 56, 58, 60–62, 64, 65, 67, 71, 73, 74, 76, 77, 80, 81] and 39% (*n* = 25) did not explicitly define sustainability [22–26, 30, 37, 38, 40, 41, 51, 52, 55, 57, 59, 63, 66, 68–70, 72, 75, 78, 79, 82]. Of the 39 studies with a clear definition, 66% (*n* = 26) drew upon one empirical source to define sustainability [20, 28, 29, 31–36, 39, 42–44, 46, 47, 50, 53, 54, 56, 60, 64, 65, 73, 74, 77, 80], 26% (*n* = 10) drew upon multiple sources [19, 27, 45, 48, 49, 58, 62, 71, 76, 81], and 8% (*n* = 3) [21, 61, 67] developed their own definitions for sustainability. The sources of sustainability definitions used in the included are detailed in Table 5. The most reported terms used to describe sustainability were continuation, maintenance, integration, routinization, normalization, and institutionalization.

**Table 5:** Sustainability definition sources

***Defining author***	^a^***Number of studies***	***Reference(s)***
Shediac-Rizkallah and Bone (1998)	5	[19, 27, 46, 48, 76]
National Health Services Sustainability Model (2010)	5	[20, 32, 54, 58, 74]
Stirman et al. (2012)	5	[19, 34, 36, 49, 50]
Scheirer and Dearing (2011)	5	[19, 33, 35, 58, 62]
May et al. (2009)	4	[42, 73, 77, 80]
Scheirer (2005)	4	[27, 48, 49, 76]
Pluye et al. (2004)	3	[27, 45, 49]
Schell, Luke, and Schooley (2013)	2	[60, 81]
Proctor et al. (2011)	2	[49, 65]
Gruen et al. (2008)	2	[27, 56]
Scheirer (2013)	2	[28, 81]
Fleiszer et al. (2015)	2	[49, 64]
Chambers et al. (2013)	1	[45]
Honadle and Sante (1985)	1	[53]
Organization for Economic Co-operation	1	[31]
Appleby et al. (2005)	1	[43]
World Health Organization (2002)	1	[29]
Own definition	3	[21, 61, 67]

^a^Total number is greater than 39 as some studies used multiple sources to define sustainability

### Theoretical approaches used to evaluate sustainability

Of the 64 studies, 44% (*n* = 28) reported that they used a framework as their primary theoretical approach to evaluate sustainability [19–21, 26, 29–31, 34–36, 39, 43–52, 55, 57, 59, 61, 63, 75, 82]. The next most common theoretical approach was a model, used in 26% (*n* = 17) of included studies [22–24, 27, 32, 33, 37, 38, 54, 56, 58, 64, 66, 72, 74, 76, 78]. A tool was the primary theoretical approach in 11% (7/64) of the included studies [60, 62, 68, 69, 71, 80, 81]. Only 5% (3/64) of included studies used an instrument, making this the least common theoretical approach used [65, 67, 77]. Theory was used as the primary theoretical approach to evaluate sustainability in 14% (9/64) of studies [25, 28, 40–42, 53, 70, 73, 79] (Table 6).

**Table 6:** Primary theoretical approaches used to evaluate sustainability

Theoretical approach		Referenced studies
**Frameworks**	**44% (*n*** **= 28)**	[19–21, 26, 29–31, 34–36, 39, 43–52, 55, 57, 59, 61, 63, 75, 82]
Single	*n* = 23	[19–21, 26, 29–31, 34–36, 39, 43, 46–50, 57, 59, 61, 63, 75, 82]
Multiple	*n* = 4	[45, 51, 52, 55]
Own	*n* = 1	[44]
**Models**	**26% (*n*** **= 17)**	[22–24, 27, 32, 33, 37, 38, 54, 56, 58, 64, 66, 72, 74, 76, 78]
Single	*n* = 7	[24, 27, 32, 33, 37, 64, 72]
Single + theory	*n* = 1	[38]
Single + tool	*n* = 5	[54, 58, 66, 74, 78]
Multiple + theory	*n* = 1	[56]
Own	*n* = 3	[22, 23, 76]
**Theory**	**14% (*n*** **= 9)**	[25, 28, 40–42, 53, 70, 73, 79]
**Instrument**	**5% (*n*** **= 3)**	[65, 67, 77]
**Tool**	**7% (*n*** **= 7)**	[60, 62, 68, 69, 71, 80, 81]

Of the 28 studies that used a framework, 82% (*n* = 23) used a single framework [19–21, 26, 29–31, 34–36, 39, 43, 46–50, 57, 59, 61, 63, 75, 82], while 14% (*n* = 4) used a combination of frameworks [45, 51, 52, 55]. The remaining 4% (*n* = 1) developed their own framework [44] to evaluate sustainability. A wide range of frameworks were used to evaluate sustainability; the Consolidated Framework for Implementation Research (CFIR [83]) was used most frequently (*n* = 5) [20, 30, 36, 61, 82], followed by the Promoting Action on Research Implementation in Health Services (PARiHS) Framework (*n* = 3) [26, 46, 63]. A total of 17% (*n* = 11) studies used a combination of theoretical approaches, as opposed to a single theoretical approach [38, 45, 51, 52, 54–56, 58, 66, 74, 78].

Of the 17 studies that used a model, 42% (*n* = 7) used a single model [24, 27, 32, 33, 37, 64, 72]. On the contrary, 18% (*n* = 3) developed their own model [22, 23, 76], whereas 9% (*n* = 1) used a combination of models with a theory for data collection purposes [56]. The National Health Service Sustainability Model (NHS SM) [84] was combined with Normalization Process Theory (NPT) [85] to inform a realist evaluation on sustainability in 9% (*n* = 1) of included studies, making this the only study combining a single model combined with a single theory [38]. The remaining 29% (*n* = 5) used a model combined with a tool for data collection purposes [54, 58, 66, 74, 78]. The NHS SM [84] was the most frequently used model to evaluate sustainability (35%; *n* = 6) [32, 54, 58, 74, 76, 78]. Of the six studies that used the NHS SM [84], five also used the NHS SM [84] as a basis for a survey for data collection [54, 58, 74, 76, 78]. One study that used the NHS SM [84] as its primary evaluation approach also drew upon the Theoretical Domains Framework [86] to develop their interview guide [58].

The Program Sustainability Assessment Tool (PSAT) [87] was the most frequently reported tool among the seven studies using a tool as the primary theoretical approach to evaluate sustainability (71%; *n* = 5) [60, 62, 69, 71, 81]. Of the 64 included studies, 5% (*n* = 3) used an instrument as their primary theoretical approach to evaluate sustainability [65, 67, 77]. Instruments used include the Technology Adoption Readiness Scale (TARS) [77] (*n* = 1) [77], Individual Placement and Support Fidelity Scale (IPS-25) [88] (*n* = 1) [67] and an adapted version of the Level of Institutionalization (LoIn) [89] Scales (*n* = 1) [65].

A total of 14% (*n* = 9) used a theory-informed process as their primary theoretical approach to evaluate sustainability, with 89% (*n* = 8) [25, 28, 40–42, 70, 73, 79] of those drawing on (NPT) [85] and 11% (*n* = 1) [53] drawing on Diffusion of Innovation Theory [90]. All the approaches are outlined in Table 7.

**Table 7:** Primary approach for evaluation of sustainability

***Approach***	***Name of primary theoretical approach used***	***Total n =***
Framework **(*n*** **= 28)**	Exploration, Preparation, Implementation, Sustainment (EPIS) framework [91] Consolidated Framework for Implementation Research [83] (CFIR) Promoting Action on Research Implementation in Health Services (PARiHS) [92] Framework Framework for investigating the sustainability of Antiretroviral (ARV) provision [21] Reach, Effectiveness, Adoption, Implementation, and Maintenance (RE-AIM) framework [93] The health technology adoption, Non-adoption, Abandonment, and challenges to Scale-up, **S**pread and Sustainability (NASSS) framework [94] Dynamic Sustainability Framework (DSF) [95] Own framework Direction, Competency, Opportunity and Motivation (DCOM®) framework [96] Framework for Sustainability of Translational Research Project [35] Conceptual framework on sustainability of community-based programs [16, 97]. Scheirer’s framework for program sustainability [16] Conceptual framework by Pomey et al. (2009) [98] Program Sustainability Framework Combination of several theoretical frameworks	(*n* = 1) (*n* = 6) (*n* = 3) (*n* = 1) (*n* = 3) (*n* = 1) (*n* = 2) (*n* = 1) (*n* = 1) (*n* = 1) (*n* = 2) (*n* = 1) (*n* = 1) (*n* = 3) (*n* = 3)
Model **(*n*** **= 17)**	National Health Service Sustainability Model (NHS SM) [84] Developed own model The British National Health Service Sustainability Index (SI) model [84] Stages of Change Model [99] Sustainability Pyramid Model [24] Mancini and Marek’s Model of Community-Based Program Sustainability [100] Kotter's 8 Steps Process for Leading Change Model [101] Dynamic model of health program sustainability [102] The Evidence in the Learning Organization (ELO) Model [103]	(*n* = 6) (*n* = 3) (*n* = 2) (*n* = 1) (*n* = 1) (*n* = 1) (*n* = 1) (*n* = 1) (*n* = 1)
Tool **(*n*** **= 7)**	Program Sustainability Assessment Tool (PSAT) [87] Sustainability Index and Dashboard Tool [68] NHS Institute for Innovation and Improvement SM Self-Assessment Tool [104]	(*n* = 5) (*n* = 1) (*n* = 1)
Theory **(*n*** **= 9)**	Diffusion of Innovation theory [90] Normalization Process Theory (NPT) [85]	(*n* = 1) (*n* = 1)
Instrument **(*n*** **= 3)**	Technology Adaption Readiness Scale (TARS) [77] Adapted Level of Institutionalization (LoIn) scales [88] Individual Placement and Support Fidelity Scale (IPS-25) [89]	(*n* = 1) (*n* = 1) (*n* = 1)

The ways in which the selected sustainability approaches were applied in the included evaluation studies fell into three categories: (1) data collection (construct measures and outcomes), (2) data analysis (to examine and interpret data in relation to sustainability); and (3) a combination of data collection and analysis.

### Research design and methodological quality

The research designs and MMAT [18] quality appraisal scores of the included studies are presented in Table 8. The scale ranges from 100 (highest quality) to 0% (lowest quality); however, all included studies ranged from 100 to 40%. More than half (59%; *n* = 38) of included studies received a quality appraisal score of 100%, indicating the included studies are of high methodological quality. Further, none of the included studies received a quality appraisal score of 0 or 20%. This is especially reflective of the high methodological quality as mixed-method studies were scored using both qualitative and quantitative descriptive categories and assigned the lower score. The rationale for this scoring supported the notion that a study can only be as strong as its weakest component [18].

**Table 8:** Study design and MMAT score of 64 included studies

Study design	Number of studies (%)	MMAT score distribution					
		0	20	40	60	80	100
Mixed-methods	26.5 (*n* = 17)	0		2	2	4	24
Multi-methods	12.5 (*n* = 8)	0		0	1	1	5
Qualitative	50 (*n* = 32)	0		1	4	6	6
Quantitative	11 (*n* = 7)	0		2	2	1	3
**Total**	**100 (*n*** **= 64)**	**0 (*n*** **= 0)**		**7.8 (*n*** **= 5)**	**14.0 (*n*** **= 9)**	**18.8 (*n*** **= 12)**	**59.4 (*n*** **= 38)**

### Reported timing of evaluation

Of 64 included studies, 66% (*n* = 43) [20, 22, 24, 25, 28, 31, 34, 35, 39–42, 44, 45, 48–59, 63, 65–76, 79–82] had a clear timing for evaluation and 33% (*n* = 21) [19, 21, 23, 26, 27, 29, 30, 32, 33, 36–38, 43, 46, 47, 60–62, 64, 77, 78] had unclear timing. Of the 43 studies with clear timing, 42% (*n* = 18) evaluated sustainability at a single time point [20, 24, 28, 34, 39–41, 44, 45, 48, 51, 53, 66, 67, 73, 80–82]. The remaining 57% (*n* = 25) evaluated sustainability at multiple time points [22, 25, 31, 35, 42, 49, 50, 52, 54–59, 63, 65, 68–72, 74–76, 79]. The majority of studies (63%; *n* = 40) conducted data collection post evaluation only [20, 22, 24, 25, 28, 31, 34, 35, 40–42, 44, 45, 48–57, 59, 63, 65–69, 71–76, 79–82]. Evaluation timing and data collection time points are provided in Table 9.

**Table 9:** Reported timing of evaluation

Timing of evaluation	Number of studies (***n*** = 64)	Points of data collection	Reference(s)
Pre- and post-intervention	*n* = 2	➔ Baseline ^a^, 1 year post, 2 years post	[59]
		➔ Baseline, 6–8 months post, 36–42 months post	[63]
Pre- and during intervention	*n* = 1	➔ Baseline, 3 years post	[58]
Pre-, during, and post-intervention	*n* = 2	➔ Baseline, 3–6 months post, 7–12 months post	[25]
		➔ 0, 1 year, 2–4 years	[68]
During intervention	*n* = 1	➔ Over 1 year: followed implementation as it occurred	[70]
During and post-intervention	*n* = 1	➔ 1 year post-training, 2 years post-training, 4 years post-training	[79]
Post-intervention	*n* = 36	➔ Under 2 years post-intervention (*n* = 15)	[24, 28, 40, 41, 51–55, 66, 69, 71, 73, 75, 80]
		➔ 2 years post-intervention (*n* = 6)	[22, 34, 45, 50, 67, 82
		➔ 3 years post-intervention (*n* = 6)	20, 35, 48, 56, 74, 76]
		➔ 4 years post-intervention (*n* = 2)	[44, 81]
		➔ 5 years post-intervention (*n* = 3)	[31, 57, 72]
		➔ 6 years post-intervention (*n* = 2)	[49, 65]
		➔ 10 + years post-intervention (*n* = 2)	[39, 42]
Unclear	*n* = 21	➔ Unclear	[19, 21, 23, 26, 27, 29, 30, 32, 33, 36–38, 43, 46, 47, 60–62, 64, 77, 78]

^a^Describes a measurement taken at a time with no intervention taking place and can be used as a benchmark to compare outcomes once the intervention begins

### Reported sustainability outcomes

We extracted and synthesized nine sustainability outcomes of the 64 included studies [6].

The majority of included studies (88%; *n* = 56) reported one or more evaluated outcome of sustainability [19–33, 35–39, 41–46, 48–53, 55–57, 59–65, 67, 68, 70–74, 76–82]. Half of these (50%; *n* = 28) were qualitative in design [19–32, 35–39, 41–46, 48, 55, 65]. One quarter (25%; *n* = 14) used a mixed-methods research design [33, 49–53, 56, 57, 59–63, 82], (13%; *n* = 7) used a multi-method design [67, 68, 70–74], and (13%; *n* = 7) used a quantitative research design [64, 76–81]. Of the 8 studies that did not report any sustainability outcomes 25%; (*n* = 2) used a mixed methods design [54, 58], 25%; (*n* = 2) used a multi-method design [66, 69], 38%; (*n* = 3) used a qualitative design [34, 40, 47], and the final 12% (*n* = 1) used a quantitative design [75].

Whether or not benefits continued for patients, staff, and stakeholders was the most commonly evaluated outcome of sustainability (*n* = 45). The most frequently reported sustained benefits are improved health outcomes and improved quality of care. For example, Blanchet et al., reported that eye care was not available prior to the implementation of the EBI, and therefore the sustained benefit to patients has been substantial [53]. Furthermore, Campbell et al. reported an absolute increase in long-term smoking cessation as an outcome of EBI sustainability [27]. Continuation of initiative activities or components of the intervention was an evaluated outcome of EBI sustainability in 36 of the included studies. Examples of sustained EBI activities include the continuation of HIV rapid testing [72], or continued use of the intervention guidebook [41]. Continuation of initiative activities or components differs from the maintenance of policies and procedures, which was only reported in 31 of the included studies. For example, Spassiani et al., reported continued EBI activities such as providing nutritious food at gatherings, and hosting community outings on evenings and weekends as an outcome of sustainability, but reported no formal policy or procedure change [46]. Alternatively, Kennedy et al. reported sustained changes in nutrition policies as well as sustained activities like giving healthy snacks, and distributing Healthy Habit Questionnaires [59].

Maintenance of relationships, partnerships or networks was a common outcome of sustainability, and reported in 34 of the included studies. The next most reported outcomes are the capacity built within staff stakeholders, and communities (*n* = 29), and adaptations made in response to new evidence or contextual influences (*n* = 29). Examples of sustained increased capacity include hiring new staff to help deliver EBI activities [24], funding a new electronic medical record system [24], and regular training [23].

Sustaining increased awareness of the issue, and replication or scale-up of the initiative, were both reported as an outcome of sustainability in 18 of the included studies. While a relatively frequent outcome of sustainability was increased attention and awareness of the problem or issue, it should be noted this was not always a good thing. For example, the increased attention around the provision of HIV treatment resulted in system capacity overload as an increased number of people sought treatment [49].

**Fig. 2:**
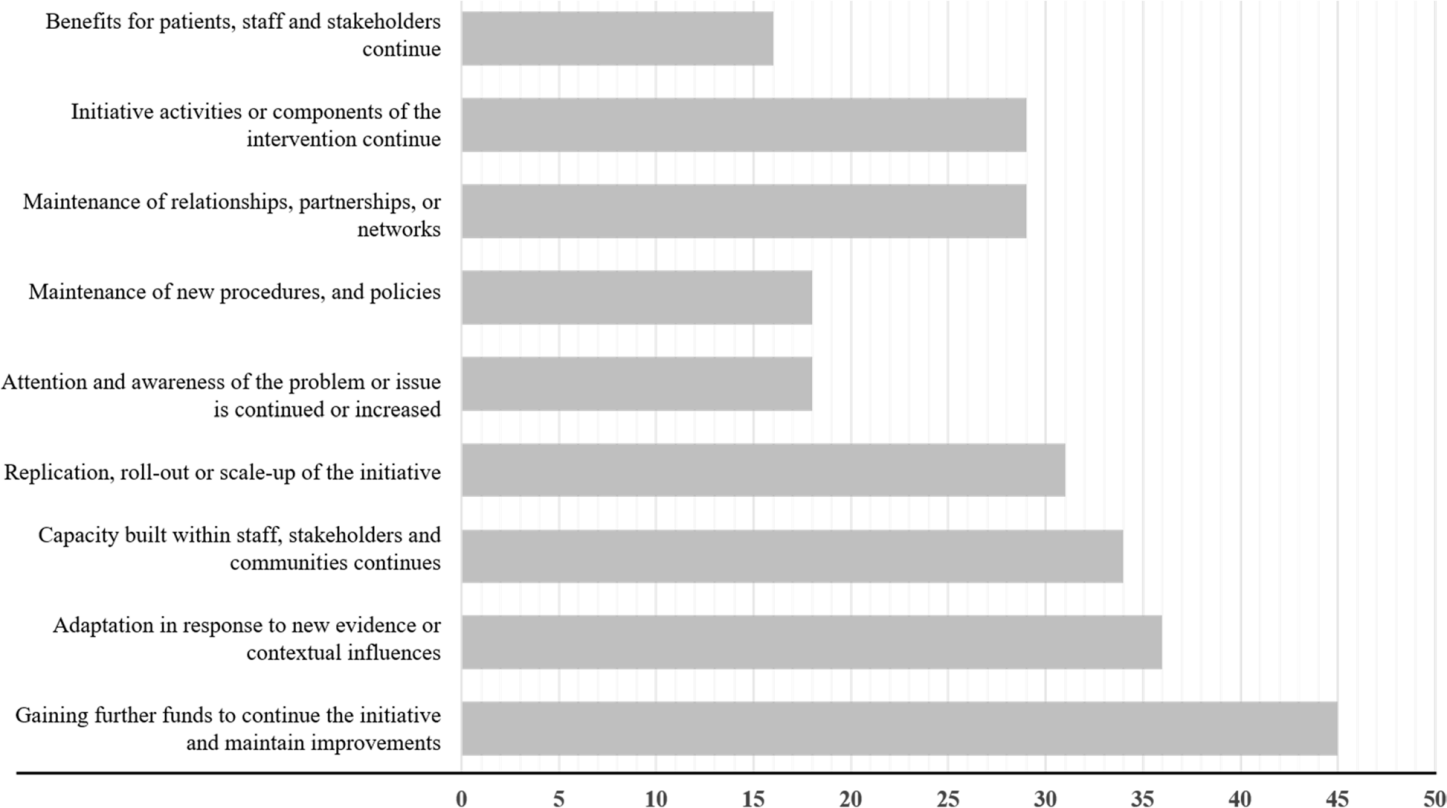
Sustainability outcomes measured

Gaining further funds to continue the initiative and maintain improvements was the least reported outcome (*n* = 16) It is apparent that funding of EBIs is often focused on implementation efforts and rarely permanent. Figure 2 depicts the distribution of reported sustainability outcomes. The outcomes reported in each of the 64 included studies can be found in Additional file 4.

## Discussion

As evaluation is necessary to determine effectiveness, this review aimed to improve our understanding of how the sustainability of EBIs in healthcare is evaluated. A focus on evaluation is what differentiates this review from recent syntheses published on approaches to sustainability research. We also need to understand how, if, and why EBIs work or not in certain contexts to enable replication, sustainability, spread, and scale [105]. Therefore, we provide evidence on theoretical approaches used for evaluation, including how and when they have been used, and offer new guidance and synthesis on the combination of approaches for evaluating sustainability of EBIs. Primary research to compare theoretical approaches used in sustainability evaluation research, to which determinants are most pertinent to sustainability evaluation is non-existent. It remains unknown the similarities and differences between these theoretical approaches. Such evidence would be highly beneficial to healthcare leaders who need guidance on what theoretical approach to select to evaluate the sustainability of an EBI they are implementing in clinical environments and not under a research effectiveness trial design. It would also be useful for researchers so that they can inform healthcare leaders on actionable evidence.

While evidence on theoretical approaches exist, we provide further insight by reporting when sustainability evaluations were performed (timing), and what methods, measures, and outcomes were reported in evaluations on the sustainability of EBIs in healthcare. We found 64 studies in the peer-review literature that used a theoretical approach to evaluate the sustainability of EBIs in healthcare. Our synthesis indicated that there is a breadth of theoretical approaches and constructs that were considered for the evaluation of the sustainability of EBIs in healthcare that were consistent with other recent synthesis work [6–10]. A recent scoping review and theory analysis [8] found 37 sustainability determinant factors, which grouped into seven themes: (1) characteristics of the innovation**/**EBP; (2) adopter/user factors influencing sustained use (3) leadership and management influences/factors; (4) inner context (practice setting/organization) factors where EBPs are delivered; (5) inner processes**/**infrastructure factors that support the EBPs (e.g., processes, methods, systems, structures, or strategies); (6) outer context or broader system factors; and (7) outcomes descriptions without defined factors. These themes are similar to the work of Lennox et al. [9], who found six themes of sustainability constructs that aligned with the five domains associated with effective implementation outlined CFIR [83]: (1) intervention characteristics; (2) outer setting; (3) inner setting; (4) characteristics of individuals; and (5) process [9].

Despite these scientific advancements on sustainability determinants, there is a lack of guidance on how to select the most appropriate theoretical approach to evaluate the sustainability of EBIs in healthcare. Interestingly, our review provides insight into the combination of theoretical approaches (e.g., theory and a tool) used to evaluate sustainability. We identified eleven studies (17% of included) that used a combined of theoretical approach, as opposed to a single theoretical approach [38, 45, 51, 52, 54–56, 58, 66, 74, 78], with the most common combination being a single model with a single tool (*n* = 5) [54, 58, 66, 74, 78]. Some theoretical approaches originated from implementation science (e.g., CFIR [83], RE-AIM [93], PARiHS [92]), where sustainability is viewed as an outcome of implementation whereas other theoretical approaches were specific to sustainability and encompass the process of sustainability and or factors that influence sustainability (e.g., NHS SM [84], DSF [95]).

Most evaluations in this integrative review applied determinant theoretical approaches, that focus on predictors of sustainability (organizational, contextual, human, and processes), but they did not link these determinants to, and, or measure patient or system level outcomes, such as sustained patient or staff benefit [6]. For those studies that did measure any of the nine sustainability outcomes, there was a lack of correlation between the outcome (e.g., maintenance of policy) and long-term impact on patient or system outcomes. In a review by Lennox et al., they indicated that only 21% of studies reported any information on sustainability outcomes [6].

Most sustainability evaluations included in this study used qualitative research designs (48%), with interviews as the most common data collection method. This finding is consistent with the work of Lennox [6] who reported 59% of their included studies used qualitative methods. While qualitative research designs gather rich detail on potential determinants of sustainability (e.g., context) and participant’s perspectives on the sustainability process, this design cannot solely measure mechanisms and outcomes of sustainability. Researchers must also consider a mixed-methods approach for sustainability evaluation. Mixed-methods research designs allow studying a phenomenon from different perspectives and provide a richer picture by combining insights from qualitative data with quantitative data [56]. The combination of quantitative and qualitative approaches is consistent with a better understanding of complex phenomena than either approach alone [57]. Our findings also highlight a significant knowledge gap on the timing of evaluation in sustainability research—there is no guidance on this matter. Almost all included studies collected sustainability data post-intervention without any pre-intervention or during intervention data collection. Evaluation of sustainability pre-intervention can help to better understand the contextual factors that may hinder or facilitate the likelihood of sustaining a particular intervention in a specific context. In our review, pre-intervention evaluations were only conducted in 6% (*n* = 4) of included studies [25, 59, 64, 68]. Of these, half (*n* = 2) used a mixed-method research design, combining semi-structured interviews with quantitative surveys and reviews [59, 64]. One study used a multi-method design including group interviews and health facility assessments [68], and the final qualitative study relied solely on interviews [25]. Timing of sustainability evaluation should be considered in relation to what was being implemented (i.e., an EBI that has been proven to be effective) and how it is being implemented (i.e., hybrid type III research design).

Of the 42 studies with clear evaluation timing, there was no clear pattern of data collection time points. Only 24 studies had multiple data collection time points. Multiple time points are necessary where feasible to adjust for the adaptation of the intervention and context over time. Measuring outcomes at multiple time points over a more extended period is also important to determine continued benefit and impact on patient care and service delivery. Such evidence would also support the sustainability of the EBI in practice [35].

Based upon the findings of our review we can offer some key methodological guidance for evaluations of the sustainability of EBIs in healthcare. Firstly, we recommend where feasible to use a combination of approaches for evaluating sustainability of EBIs. A combination of approaches that can evaluate sustainability determinant and outcomes will facilitate our understanding of linkages between determinants and patient or system level outcomes. Secondly, we recommend mixed-methods approach for sustainability evaluation. Mixed methods research designs can provide a better understanding of complex phenomena. Thirdly, we recommend evaluations of sustainability at multiple time points, including pre-intervention in order understand the evolution of sustainability over time. Finally, we recommend that future research is needed to understand mechanisms of sustainability to advance the field. From our review, these mechanisms have not yet been identified for sustainability. There is evidence on determinants for sustainability, and outcomes for sustainability but there is a knowledge gap on how and why under what contexts certain determinants lead to specific outcomes. Mechanisms are underlying entities, process, or structures which operate in particular contexts to generate outcomes of interest [106]. Mechanisms offer causal pathways to understand how and why, under what contexts a determinant of sustainability does or does not achieve its intended effect. This knowledge will advance researchers and health system implementers ability to design, develop and implement strategies that directly target sustainability determinants and outcomes.

## Limitations

We only included published studies in the English language and peer-reviewed primary studies in this review. This review entailed a comprehensive search of published literature and rigorous review methods; however, we recognize that there is the possibility of incomplete retrieval of identified research. For example, all gray literature was excluded.

## Conclusions

Our review has emphasized areas that require further research and the need for methodological guidance for sustainability evaluations of EBIs in healthcare. Advancing our understanding in this area would facilitate better design and tailored strategies for sustainability, therefore contributing to the success of sustainability efforts. This work contributes to existing syntheses on sustainability approaches, specifically for evaluation research and on ways to move forward to advance this field.

## Supplementary Information

**Additional file 1. **Search strategy. The four databases searched on Dec 21, 2018, as well as an additional search in July 2020. The file depicts the date of searchers and strategy used, including all search terms in each database.

**Additional file 2.** PRISMA 2020 Checklist. The complete 2020 PRISMA checklist for new systematic reviews.

**Additional file 3. **Citation list of included studies. A citation list of all included studies (*n*=64). They are listed in alphabetical order, according to the Vancouver referencing style.

**Additional file 4. **Citation list of included studies. A table of the sustainability outcomes reported in all of the included studies (*n*=64). The table depicts which of the 9 sustainability outcomes were reported in each study.

## Data Availability

The datasets used and/or analyzed during the current study are available from the corresponding author on reasonable request.

## Abbreviations

CFIR: Consolidated Framework for Implementation Research
DSF: Dynamic Sustainability Framework
EBI: Evidence-based intervention
EBP: Evidence Based Practice
IPS-25: Individual Placement and Support Fidelity Scale
IS: Implementation Science
LoIn: Scales Level of Institutionalization Scales
MMAT: Mixed Methods Appraisal Tool
NHS SM: The National Health Service Sustainability Model
NPT: Normalization Process Theory
PARiHS: Promoting Action on Research Implementation in Health Services Framework
PRISMA: Preferred Reporting Items for Systematic Reviews and Meta-analyses
PSAT: Program Sustainability Assessment Tool
RE-AIM: Reach, Effectiveness, Adoption, Implementation, and Maintenance
TARS: Technology Adaption Readiness Scale
