# Supplementary material for: Identifying existing approaches used to evaluate the sustainability of evidence-based interventions in healthcare: an integrative review
Source: Syst Rev. 2022 Oct 15;11:221. doi: 10.1186/s13643-022-02093-1 (PMC9569065; doi:10.1186/s13643-022-02093-1)
Supplement: Supplementary file 1 — Additional file 1. Search strategy. The four databases searched on Dec 21, 2018, as well as an additional search in July 2020. The file depicts the date of searchers and strategy used, including all search terms in each database. [file 13643_2022_2093_MOESM1_ESM.docx]

**Additional File 1. Search Strategy**

**Sustainability**

The following databases were searched on Dec 21, 2018

- Ovid MEDLINE(R) and Epub Ahead of Print, In-Process & Other Non-Indexed Citations and Daily <1946 to Current>
- Embase <1974 to Current>
- EBSCO CINAHL Plus with Full-text, 1937-Current
- Wiley Cochrane Library, inception to current

Terms representing sustainability were combined with terms representing knowledge translation/implementation of healthcare programs and terms related to evaluation/measurement.  A set of terms was created to exclude irrelevant studies as far as possible without eliminating relevant studies, e.g. terms representing environmental sustainability, patient institutionalisation, animal studies, etc.  Results from this set were excluded from the main set of results.

Results were limited to English language and to academic journals (when the interface permitted).

13,613 items were retrieved in total. 8,214 remained after duplicates were removed.

An update of the search was performed using the same original databases and search strategies in July 2020. 5170 new items were found in total. 2452 items remained after duplicates were removed.

**Ovid MEDLINE(R) and Epub Ahead of Print, In-Process & Other Non-Indexed Citations and Daily <1946 to July 23, 2020>**

--------------------------------------------------------------------------------

1     (program* adj2 sustain*).ti,kf. (274)

2     (sustain* or institutionali* or routini* or normali?ation).ti. or ((sustain or sustainab* or sustaining or institutionali* or routini* or normali?ation) adj5 (program* or service* or system or quality or culture or evidence or assess* or measur* or evaluat* or indicator* or analys* or model* or framework* or theor*)).ab. (58085)

3     *translational medical research/ or *diffusion of innovation/ or *quality improvement/ or *models, organizational/ or *health impact assessment/ or *program planning/ or *health services research/ (42963)

4     (((translat* or implement* or adopt* or uptake or scale) and (change or improv* or research or knowledge or innovat* or evidence or outcome*)) or ((change or improv* or implement*) adj3 (manag* or strateg* or success*)) or ((quality adj1 improv*) or knowledge translation or evidence-based)).ti,ab,kf. or (chang* or improv*).ti. (1588877)

5     *Organizational Innovation/ or exp Health Services Accessibility/ or og.fs. or (((health or patient*) adj2 care) or healthcare or hospital*).ti,ab,kf. (1986706)

6     3 or 4 or 5 (3312420)

7     "surveys and questionnaires"/ or program evaluation/ or Cost-Benefit Analysis/ or (tool or scale or instrument or index or battery or survey* or questionnaire* or checklist*).ti,ab,kf. or ((measur* or assess* or evaluat* or score* or analys*) adj4 (sustain* or adopt* or implement* or impact*)).ti,ab,kf. or (measur* or evaluat* or assess* or value or valuable).ti,kf. or (determin* adj2 level*).ti,ab,kf. or *"delivery of health care"/st or exp Health Services/st or exp *"Outcome and Process Assessment (Health Care)"/ (3902272)

8     2 and 6 and 7 (8118)

9     1 and 7 (193)

10     8 or 9 (8210)

11     animals/ or (veterinar* or zoo or zoos or species or habitat* or environment* or conservation or insect* or land management or "land use" or pollution or water or wastewater or soil or biodiesel or biofuel or urban or air quality or clean air or agricultur* or climate change or global warming or life sustaining or (institutionali?ed adj3 (people or person* or patient* or elderly))).mp. (8367156)

12    ((sustained adj2 (release or attention or remission or sleep or control or response or decrease* or increase*)).ti. not (sustainab* or sustainment).mp.) or (sustain* adj3 (injur* or wound*)).mp. (18557)

13     11 or 12 (8381814)

14     10 not 13 (5168)

15   ((sustainab* or institutionali* or normali* or routini*) adj (scale or tool or checklist or index or instrument)).ti,ab,kf. (342)

16     14 or 15 (5491)

17     limit 16 to english language (5279)

18     limit 17 to (comment or editorial or letter or "review") (537)

19     17 not 18 (4742)

20     limit 17 to meta analysis (17)

21     ((systematic or scoping) adj2 (review* or overview*)).ti,ab,kf. (142141)

22     17 and 21 (167)

23     19 or 20 or 22 (4850)

24     remove duplicates from 23 (4841)

**Embase <1974 to 2020 July 23 >**

--------------------------------------------------------------------------------

1     (program* adj2 sustain*).ti,kw. or program sustainability/ (1162)

2     (sustain* or institutionali* or routini* or normali?ation).ti. or (((sustain or sustainab* or sustaining or institutionali* or routini* or normali?ation) adj5 (program* or service* or system or quality or culture or evidence or assess* or measur* or evaluat* or indicator* or analys* or model* or framework* or theor*)) or long term success).ab. (80495)

3     *translational research/ or *total quality management/ or *organization/ or *"organization and management"/ or *organizational development/ or *organizational efficiency/ or *organizational theory/ or exp *health care delivery/ or *Health care practice/ or *health program/ or *program development/ or exp *health care/ or *health service/ (1459864)

4     (((translat* or implement* or adopt* or uptake or scale) and (change or improv* or research or knowledge or innovat* or evidence or outcome*)) or ((change or improv* or implement*) adj3 (manag* or strateg* or success*)) or ((quality adj1 improv*) or knowledge translation or evidence-based)).ti,ab,kw. or (chang* or improv*).ti. (2019357)

5     (((health or patient*) adj2 care) or healthcare or hospital* or health facilit*).ti,ab,kw. (2315170)

6     3 or 4 or 5 (4916500)

7     2 and 6 (26408)

8     *questionnaire/ or *open ended questionnaire/ or *structured questionnaire/ or exp *program evaluation/ or *economic evaluation/ or *health services research/ or *Performance measurement system/ or *health impact assessment/ or *outcome assessment/ (75808)

9     (tool or scale or instrument or index or battery or survey* or questionnaire* or checklist* or ((measur* or assess* or evaluat* or score* or analys*) adj4 (sustain* or adopt* or implement* or impact* or value or success*))).ti,ab,kw. or (measur* or evaluat* or assess* or value or valuable).ti,kw. or (determin* adj2 level*).ti,ab,kw. (4788595)

10     8 or 9 (4813450)

11     7 and 10 (11536)

12     1 and 10 (588)

13     11 or 12 (11802)

14     (veterinar* or zoo or zoos or species or habitat* or environment* or conservation or insect* or land management or "land use" or pollution or water or wastewater or soil or biodiesel or biofuel or urban or air quality or clean air or agricultur* or climate change or global warming or life sustaining or (institutionali?ed adj3 (people or person* or patient* or elderly))).mp. (3566619)

15     ((sustained adj2 (release or attention or remission or sleep or control or response or decrease* or increase*)).ti. not (sustainab* or sustainment).mp.) or (sustain* adj3 (injur* or wound*)).mp. (25134)

16     14 or 15 (3589321)

17     13 not 16 (7660)

18     ((sustainab* or institutionali* or normali* or routini* or long term success) adj (scale or tool or checklist or index or instrument)).mp. (472)

19     17 or 18 (8103)

20     limit 19 to english language (7819)

21     limit 20 to (conference abstract or "conference review" or editorial or letter or note or "review") (3196)

22     20 not 21 (4623)

23    ((systematic or scoping or integrative) adj2 (review or overview)).ti,ab,kw. (166583)

24    20 and 23 (182)

25    22 or 24 (4745)

26    remove duplicates from 25 (4622)

**EBSCO CINAHL Plus with Full-text**

**Date of search: July 24, 2020**

S1 (program* n2 sustain*)

S2  TI ( sustain* or institutionali* or routini*  or normalisation or normalization ) OR AB ( (sustain or sustainab* or sustaining or institutionali* or routini* or normalisation or normalization) n4 (program* or service* or system or quality or culture or evidence or assess* or measur* or evaluat* or indicator* or analys* or model* or framework* or theor*) )

S3  (MH "Diffusion of Innovation") OR (MH "Quality Improvement") OR (MH "Organizational Objectives") OR (MH "Health Impact Assessment") OR (MH "Program Planning") OR (MH "Health Services Research+") OR (MH "Health Services Accessibility+")) OR ((translat* or implement* or adopt* or uptake or scale) and (change or improv* or research or knowledge or innovat* or evidence or outcome*) ) OR ( (change or improv* or implement*) n3 (manag* or strateg* or success*) ) OR quality n2 improv* or "knowledge translation" or "evidence-based"   OR (health or patient*) w2 care or healthcare or hospital* OR TI ( chang* or improv* )

S4  S2 AND S3

S5  S1 OR S4

S6 ( (MH "Surveys") OR (MH "Questionnaires+") OR (MH "Program Evaluation") OR (MH "Cost Benefit Analysis") OR (MH "Health Care Delivery+/ST") OR (MH "Health Services+/ST") OR (MH "Outcome Assessment") OR (MH "Quality Assessment+") OR (MH "Process Assessment (Health Care)+") ) OR ( tool or scale or instrument or index or battery or survey* or questionnaire* or checklist* ) OR ( (measur* or assess* or evaluat* or score* or analys*) n3 (sustain* or adopt* or implement* or impact*) or determin* w2 level* ) OR ( measur* or evaluat* or assess* or value or valuable )

S7  S5 AND S6

S8  veterinar* or zoo or zoos or species or habitat* or environment* or conservation or insect* or "land management" or "land use" or pollution or water or wastewater or soil or biodiesel or biofuel or urban or "air quality" or "clean air" or agricultur* or "climate change" or "global warming" or "life sustaining"  or ((institutionalized or institutionalised) w3 (people or person* or patient* or elderly)) or TI(sustained w2 (release or attention or remission or sleep or control or response or decrease* or increase*)) or ((sustain or sustained or sustains or sustaining) w2 (injur* or wound* or trauma*))

S9 S7 NOT S8

S10 ((sustainab* or institutionali* or normali* or routini*) w1 (scale or tool or checklist or index or instrument))

S11  S9 OR S10

LIMIT:  English language and Academic Journals

**Wiley COCHRANE Library**

**Date of search: July 24, 2020**

#1     (program* near/2 sustain*):ti

#2     (sustain* or institutionali* or routini* or normalization or normalisation):ti or ((sustain or sustainab* or sustaining or institutionali* or routini* or normalization or normalisation) near/5 (program* or service* or system or quality or culture or evidence or assess* or measur* or evaluat* or indicator* or analys* or model* or framework* or theor*)):ab

#3     (((translat* or implement* or adopt* or uptake or scale) and (change or improv* or research or knowledge or innovat* or evidence or outcome*)) or ((change or improv* or implement*) near/2 (manag* or strateg* or success*)) or ((quality near/2 improv*) or "knowledge translation" or "evidence-based")):ti,ab,kw or (chang* or improv*):ti

#4  #2 AND #3

#5  #1 OR #4

#6 (tool or scale or instrument or index or battery or survey* or questionnaire* or checklist*):ti,ab,kw or ((measur* or assess* or evaluat* or score* or analys*) near/4 (sustain* or adopt* or implement* or impact*)):ti,ab,kw or (measur* or evaluat* or assess* or value or valuable):ti or (determin* near/2 level*):ti,ab,kw

#7  #5 AND #6

#8 (veterinar* or zoo or zoos or species or habitat* or environment* or conservation or insect* or land management or "land use" or pollution or water or wastewater or soil or biodiesel or biofuel or urban or air quality or clean air or agricultur* or climate change or global warming or life sustaining or (sustained near/2 (release or attention or remission or sleep or control or response)) or ((sustain or sustains or sustained or sustaining) near/2 (injur* or wound* or trauma*)) or ((institutionalized or institutionalised) near/3 (people or person* or patient* or elderly))):ti,ab,kw

#9  #7 NOT #8

#10 ((sustainab* or institutionali* or normali* or routini*) near/2 (scale or tool or checklist or index or instrument))

#11  #9 OR #10

Limited to Cochrane Database of Systematic Reviews and Cochrane Central Register of Controlled Trials
