# Supplementary material for: Identifying existing approaches used to evaluate the sustainability of evidence-based interventions in healthcare: an integrative review
Source: Syst Rev. 2022 Oct 15;11:221. doi: 10.1186/s13643-022-02093-1 (PMC9569065; doi:10.1186/s13643-022-02093-1)
Supplement: Supplementary file 3 — Additional file 3. Citation list of included studies. A citation list of all included studies (n=64). They are listed in alphabetical order, according to the Vancouver referencing style. [file 13643_2022_2093_MOESM3_ESM.docx]

**Additional File 1:** Citation List of Included Studies.

1. Aarons GA, Green AE, Trott E, Willging CE, Torres EM, Ehrhart MG, et al. The Roles of System and Organizational Leadership in System-Wide Evidence-Based Intervention Sustainment: A Mixed-Method Study. Adm Policy Ment Heal Ment Heal Serv Res [Internet]. 2016 Nov 20;43(6):991–1008. Available from: http://link.springer.com/10.1007/s10488-016-0751-4
2. Abimbola S, Patel B, Peiris D, Patel A, Harris M, Usherwood T, et al. The NASSS framework for ex post theorisation of technology-supported change in healthcare: worked example of the TORPEDO programme. BMC Med [Internet]. 2019 Dec 30;17(1):233. Available from: https://bmcmedicine.biomedcentral.com/articles/10.1186/s12916-019-1463-x
3. Allchin B, Weimand BM, O’Hanlon B, Goodyear M. Continued capacity: Factors of importance for organizations to support continued Let’s Talk practice – a mixed‐methods study. Int J Ment Health Nurs [Internet]. 2020 Dec 28;29(6):1131–43. Available from: https://onlinelibrary.wiley.com/doi/10.1111/inm.12754
4. Ament SMC, Gillissen F, Moser A, Maessen JMC, Dirksen CD, von Meyenfeldt MF, et al. Factors associated with sustainability of 2 quality improvement programs after achieving early implementation success. A qualitative case study. J Eval Clin Pract. 2017;23(6):1135–43.
5. Azeredo TB, Oliveira MA, Santos-Pinto CDB, Miranda ES, Osorio-de-Castro CGS. Sustainability of ARV provision in developing countries: challenging a framework based on program history. Cien Saude Colet [Internet]. 2017 Aug;22(8):2581–94. Available from: http://www.scielo.br/scielo.php?script=sci_arttext&pid=S1413-81232017002802581&lng=en&tlng=en
6. Baloh J, Zhu X, Ward MM. What Influences Sustainment and Nonsustainment of Facilitation Activities in Implementation? Analysis of Organizational Factors in Hospitals Implementing TeamSTEPPS. Med Care Res Rev [Internet]. 2021 Apr 16;78(2):146–56. Available from: http://journals.sagepub.com/doi/10.1177/1077558719848267
7. Belizán M, Bergh A-M, Cilliers C, Pattinson RC, Voce A. Stages of change: A qualitative study on the implementation of a perinatal audit programme in South Africa. BMC Health Serv Res [Internet]. 2011 Dec 30;11(1):243. Available from: https://bmchealthservres.biomedcentral.com/articles/10.1186/1472-6963-11-243
8. Belostotsky V, Laing C, White DE. The sustainability of a quality improvement initiative. Healthc Manag Forum [Internet]. 2020 Sep 6;33(5):195–9. Available from: http://journals.sagepub.com/doi/10.1177/0840470420913055
9. Berendsen BAJ, Kremers SPJ, Savelberg HHCM, Schaper NC, Hendriks MRC. The implementation and sustainability of a combined lifestyle intervention in primary care: mixed method process evaluation. BMC Fam Pract [Internet]. 2015 Dec 17;16(1):37. Available from: https://bmcfampract.biomedcentral.com/articles/10.1186/s12875-015-0254-5
10. Blanchet K, James P. Can international health programmes be sustained after the end of international funding: the case of eye care interventions in Ghana. BMC Health Serv Res [Internet]. 2014 Dec 19;14(1):77. Available from: https://bmchealthservres.biomedcentral.com/articles/10.1186/1472-6963-14-77
11. Bond GR, Drake RE, Becker DR, Noel VA. The IPS learning community: A longitudinal study of sustainment, quality, and outcome. Psychiatr Serv. 2016;67(8):864–9.
12. Bray P, Cummings DM, Wolf M, Massing MW, Reaves J. After the Collaborative Is Over: What Sustains Quality Improvement Initiatives in Primary Care Practices? Jt Comm J Qual Patient Saf [Internet]. 2009 Oct;35(10):502-AP3. Available from: https://linkinghub.elsevier.com/retrieve/pii/S1553725009350692
13. Bridges J, May C, Fuller A, Griffiths P, Wigley W, Gould L, et al. Optimising impact and sustainability: a qualitative process evaluation of a complex intervention targeted at compassionate care. BMJ Qual Saf [Internet]. 2017 Dec;26(12):970–7. Available from: https://qualitysafety.bmj.com/lookup/doi/10.1136/bmjqs-2017-006702
14. Butow P, Williams D, Thewes B, Tesson S, Sharpe L, Smith A Ben, et al. A psychological intervention ( ConquerFear ) for treating fear of cancer recurrence: Views of study therapists regarding sustainability. Psychooncology [Internet]. 2019 Mar;28(3):533–9. Available from: https://onlinelibrary.wiley.com/doi/10.1002/pon.4971
15. Campbell S, Pieters K, Mullen K-A, Reece R, Reid RD. Examining sustainability in a hospital setting: Case of smoking cessation. Implement Sci [Internet]. 2011 Dec 14;6(1):108. Available from: http://implementationscience.biomedcentral.com/articles/10.1186/1748-5908-6-108
16. Carlfjord S, Lindberg M, Andersson A. Sustained use of a tool for lifestyle intervention implemented in primary health care: a 2-year follow-up. J Eval Clin Pract [Internet]. 2013 Apr;19(2):327–34. Available from: https://onlinelibrary.wiley.com/doi/10.1111/j.1365-2753.2012.01827.x
17. Carstensen K, Brostrøm Kousgaard M, Burau V. Sustaining an intervention for physical health promotion in community mental health services: A multisite case study. Health Soc Care Community [Internet]. 2019 Mar;27(2):502–15. Available from: https://onlinelibrary.wiley.com/doi/10.1111/hsc.12671
18. Curry SJ, Mermelstein RJ, Sporer AK. Sustainability of Community-Based Youth Smoking Cessation Programs. Health Promot Pract [Internet]. 2016 Nov 9;17(6):845–52. Available from: https://doi.org/10.1177/1524839916657326
19. Dharmayat KI. Sustainability of ‘mHealth’ interventions in sub-Saharan Africa: a stakeholder analysis of an electronic community case management project in Malawi. Malawi Med J [Internet]. 2019 Sep 3;31(3):177–83. Available from: https://www.ajol.info/index.php/mmj/article/view/191288
20. Doyle C, Howe C, Woodcock T, Myron R, Phekoo K, McNicholas C, et al. Making change last: applying the NHS institute for innovation and improvement sustainability model to healthcare improvement. Implement Sci [Internet]. 2013 Dec 26;8(1):127. Available from: http://implementationscience.biomedcentral.com/articles/10.1186/1748-5908-8-127
21. Eakin MN, Ugbah L, Arnautovic T, Parker AM, Needham DM. Implementing and sustaining an early rehabilitation program in a medical intensive care unit: A qualitative analysis. J Crit Care [Internet]. 2015;30(4):698–704. Available from: http://dx.doi.org/10.1016/j.jcrc.2015.03.019
22. El Bcheraoui C, Kamath AM, Dansereau E, Palmisano EB, Schaefer A, Hernandez B, et al. Results-based aid with lasting effects: sustainability in the Salud Mesoamérica Initiative. Global Health [Internet]. 2018 Dec 16;14(1):97. Available from: https://globalizationandhealth.biomedcentral.com/articles/10.1186/s12992-018-0418-x
23. Finch TL, Mair FS, Odonnell C, Murray E, May CR. From theory to “measurement” in complex interventions: Methodological lessons from the development of an e-health normalisation instrument. BMC Med Res Methodol. 2012;12:1–16.
24. Flynn R, Rotter T, Hartfield D, Newton AS, Scott SD. A realist evaluation to identify contexts and mechanisms that enabled and hindered implementation and had an effect on sustainability of a lean intervention in pediatric healthcare. BMC Health Serv Res. 2019;19(1):1–13.
25. Ford II JH, Alagoz E, Dinauer S, Johnson KA, Pe-Romashko K, Gustafson DH. Successful Organizational Strategies to Sustain Use of A-CHESS: A Mobile Intervention for Individuals With Alcohol Use Disorders. J Med Internet Res [Internet]. 2015 Aug 18;17(8):e201. Available from: http://www.jmir.org/2015/8/e201/
26. Ford JH, Krahn D, Wise M, Oliver KA. Measuring Sustainability Within the Veterans Administration Mental Health System Redesign Initiative. Qual Manag Health Care [Internet]. 2011 Oct;20(4):263–79. Available from: https://journals.lww.com/00019514-201110000-00003
27. Frykman M, Von Thiele Schwarz U, Athlin ÅM, Hasson H, Mazzocato P. The work is never ending: Uncovering teamwork sustainability using realistic evaluation. J Heal Organ Manag. 2017;31(1):64–81.
28. Garst J, L’Heveder R, Siminerio LM, Motala AA, Gabbay RA, Chaney D, et al. Sustaining diabetes prevention and care interventions: A multiple case study of translational research projects. Diabetes Res Clin Pract [Internet]. 2017 Aug;130:67–76. Available from: http://dx.doi.org/10.1016/j.diabres.2017.04.025
29. Graham JR, Naylor P-J. Sustainability drivers of Canada’s most health-promoting hospital. Healthc Manag Forum [Internet]. 2019 May 4;32(3):158–62. Available from: http://journals.sagepub.com/doi/10.1177/0840470418820546
30. Greenhalgh T, MacFarlane F, Barton-Sweeney C, Woodard F. “If we build it, will it stay?” A case study of the sustainability of whole-system change in London [Internet]. Vol. 90, Milbank Quarterly. Milbank Q; 2012 [cited 2021 Nov 20]. p. 516–47. Available from: https://pubmed.ncbi.nlm.nih.gov/22985280/
31. Grow HMG, Hencz P, Verbovski MJ, Gregerson L, Liu LL, Dossett L, et al. Partnering for Success and Sustainability in Community-Based Child Obesity Intervention. Fam Community Health [Internet]. 2014 Jan;37(1):45–59. Available from: <https://journals.lww.com/00003727-201401000-00007>
32. Healey J, Conlon CM, Malama K, Hobson R, Kaharuza F, Kekitiinwa A, et al. Sustainability and Scale of the Saving Mothers, Giving Life Approach in Uganda and Zambia. Glob Heal Sci Pract [Internet]. 2019 Mar 11;7(Supplement 1):S188–206. Available from: http://www.ghspjournal.org/lookup/doi/10.9745/GHSP-D-18-00265
33. Hovlid E, Bukve O, Haug K, Aslaksen AB, von Plessen C. Sustainability of healthcare improvement: what can we learn from learning theory? BMC Health Serv Res [Internet]. 2012 Dec 3;12(1):235. Available from: https://bmchealthservres.biomedcentral.com/articles/10.1186/1472-6963-12-235
34. Hunter SB, Han B, Slaughter ME, Godley SH, Garner BR. Associations between implementation characteristics and evidence-based practice sustainment: a study of the Adolescent Community Reinforcement Approach. Implement Sci [Internet]. 2015 Dec 24;10(1):173. Available from: http://dx.doi.org/10.1186/s13012-015-0364-4
35. Jones S, Hamilton S, Bell R, Araújo-Soares V, Glinianaia S V., Milne EMG, et al. What helped and hindered implementation of an intervention package to reduce smoking in pregnancy: Process evaluation guided by normalization process theory. BMC Health Serv Res. 2019;19(1):1–14.
36. Kacholi G, Mahomed OH. Sustainability of quality improvement teams in selected regional referral hospitals in Tanzania. Int J Qual Heal Care [Internet]. 2020 Jun 4;32(4):259–65. Available from: https://academic.oup.com/intqhc/article/32/4/259/5827021
37. Kastner M, Sayal R, Oliver D, Straus SE, Dolovich L. Sustainability and scalability of a volunteer-based primary care intervention (Health TAPESTRY): a mixed-methods analysis. BMC Health Serv Res [Internet]. 2017 Dec 1;17(1):514. Available from: http://bmchealthservres.biomedcentral.com/articles/10.1186/s12913-017-2468-9
38. Kempen TGH, Gillespie U, Färdborg M, McIntosh J, Mair A, Stewart D. A case study of the implementation and sustainability of medication reviews in older patients by clinical pharmacists. Res Soc Adm Pharm [Internet]. 2019;15(11):1309–16. Available from: https://doi.org/10.1016/j.sapharm.2018.12.006
39. Kennedy L, Pinkney S, Suleman S, Mâsse L, Naylor P-J, Amed S. Propagating Change: Using RE-FRAME to Scale and Sustain A Community-Based Childhood Obesity Prevention Initiative. Int J Environ Res Public Health [Internet]. 2019 Mar 1;16(5):736. Available from: https://www.mdpi.com/1660-4601/16/5/736
40. King DK, Gonzalez SJ, Hartje JA, Hanson BL, Edney C, Snell H, et al. Examining the sustainability potential of a multisite pilot to integrate alcohol screening and brief intervention within three primary care systems. Transl Behav Med [Internet]. 2018 Sep 8;8(5):776–84. Available from: https://academic.oup.com/tbm/article/8/5/776/4820868
41. Klinga C, Hasson H, Andreen Sachs M, Hansson J. Understanding the dynamics of sustainable change: A 20-year case study of integrated health and social care. BMC Health Serv Res. 2018;18(1):1–12.
42. Knapp H, Hagedorn H, Anaya HD. A five-year self-sustainability analysis of nurse-administered HIV rapid testing in Veterans Affairs primary care. Int J STD AIDS. 2014;25(12):837–43.
43. Kosse RC, Murray E, Bouvy ML, de Vries TW, Stevenson F, Koster ES. Potential normalization of an asthma mHealth intervention in community pharmacies: Applying a theory-based framework. Res Soc Adm Pharm [Internet]. 2020 Feb;16(2):195–201. Available from: https://doi.org/10.1016/j.sapharm.2019.05.004
44. Lillvis DF, Willison C, Noyes K. Normalizing inconvenience to promote childhood vaccination: a qualitative implementation evaluation of a novel Michigan program. BMC Health Serv Res [Internet]. 2020 Dec 23;20(1):683. Available from: https://bmchealthservres.biomedcentral.com/articles/10.1186/s12913-020-05550-6
45. Lindholm LH, Koivukangas A, Lassila A, Kampman O. What is important for the sustained implementation of evidence-based brief psychotherapy interventions in psychiatric care? A quantitative evaluation of a real-world programme. Nord J Psychiatry [Internet]. 2019 Apr 3;73(3):185–94. Available from: https://www.tandfonline.com/doi/full/10.1080/08039488.2019.1582698
46. Mahomed OH, Asmall S, Voce A. Sustainability of the integrated chronic disease management model at primary care clinics in South Africa. African J Prim Heal Care Fam Med [Internet]. 2016 Nov 17;8(1):1–7. Available from: https://phcfm.org/index.php/phcfm/article/view/1248
47. Morden A, Brooks L, Jinks C, Porcheret M, Ong BN, Dziedzic K. Research “push”, long term-change, and general practice. J Health Organ Manag [Internet]. 2015 Nov 16;29(7):798–821. Available from: https://www.emerald.com/insight/content/doi/10.1108/JHOM-07-2014-0119/full/html
48. Nazar H, Nazar Z. Community pharmacy minor ailment services: Pharmacy stakeholder perspectives on the factors affecting sustainability. Res Soc Adm Pharm [Internet]. 2019;15(3):292–302. Available from: https://doi.org/10.1016/j.sapharm.2018.04.036
49. Nordmark S, Zingmark K, Lindberg I. Process evaluation of discharge planning implementation in healthcare using normalization process theory. BMC Med Inform Decis Mak [Internet]. 2016 Dec 27;16(1):48. Available from: http://dx.doi.org/10.1186/s12911-016-0285-4
50. Palinkas LA, Spear SE, Mendon SJ, Villamar J, Reynolds C, Green CD, et al. Conceptualizing and measuring sustainability of prevention programs, policies, and practices. Transl Behav Med [Internet]. 2020 Feb 3;10(1):136–45. Available from: https://academic.oup.com/tbm/article/10/1/136/5640460
51. Pomey M-P, Clavel N, Amar C, Sabogale-Olarte JC, Sanmartin C, De Coster C, et al. Wait time management strategies for total joint replacement surgery: sustainability and unintended consequences. BMC Health Serv Res [Internet]. 2017 Dec 7;17(1):629. Available from: http://bmchealthservres.biomedcentral.com/articles/10.1186/s12913-017-2568-6
52. Rasschaert F, Decroo T, Remartinez D, Telfer B, Lessitala F, Biot M, et al. Sustainability of a community-based anti-retroviral care delivery model - a qualitative research study in Tete, Mozambique. J Int AIDS Soc [Internet]. 2014 Jan;17(1):18910. Available from: http://doi.wiley.com/10.7448/IAS.17.1.18910
53. Seppey M, Ridde V, Touré L, Coulibaly A. Donor-funded project’s sustainability assessment: a qualitative case study of a results-based financing pilot in Koulikoro region, Mali. Global Health [Internet]. 2017 Dec 8;13(1):86. Available from: https://globalizationandhealth.biomedcentral.com/articles/10.1186/s12992-017-0307-8
54. Smith ML, Durrett NK, Schneider EC, Byers IN, Shubert TE, Wilson AD, et al. Examination of sustainability indicators for fall prevention strategies in three states. Eval Program Plann [Internet]. 2018 Jun;68(February):194–201. Available from: https://linkinghub.elsevier.com/retrieve/pii/S0149718917302434
55. Spassiani NA, Meisner BA, Abou Chacra MS, Heller T, Hammel J. What is and isn’t working: Factors involved in sustaining community-based health and participation initiatives for people ageing with intellectual and developmental disabilities. J Appl Res Intellect Disabil. 2019;32(6):1465–77.
56. Stoll S, Janevic M, Lara M, Ramos-Valencia G, Stephens TB, Persky V, et al. A Mixed-Method Application of the Program Sustainability Assessment Tool to Evaluate the Sustainability of 4 Pediatric Asthma Care Coordination Programs. Prev Chronic Dis [Internet]. 2015 Dec 3;12(12):150133. Available from: http://www.cdc.gov/pcd/issues/2015/15_0133.htm
57. Stolldorf DP, Fortune-Britt AG, Nieuwsma JA, Gierisch JM, Datta SK, Angel C, et al. Measuring sustainability of a grassroots program in a large integrated health care delivery system: the Warrior to Soul Mate Program. J Mil Veteran Fam Heal [Internet]. 2018 Oct;4(2):81–90. Available from: https://jmvfh.utpjournals.press/doi/10.3138/jmvfh.2017-0007
58. Stolldorf DP, Mixon AS, Auerbach AD, Aylor AR, Shabbir H, Schnipper J, et al. Implementation and sustainability of a medication reconciliation toolkit: A mixed methods evaluation. Am J Heal Pharm [Internet]. 2020 Jul 7;77(14):1135–43. Available from: https://academic.oup.com/ajhp/article/77/14/1135/5864549
59. Sving E, Fredriksson L, Mamhidir A-G, Högman M, Gunningberg L. A multifaceted intervention for evidence-based pressure ulcer prevention. Int J Evid Based Healthc [Internet]. 2020 Jul 9;Publish Ah(4):391–400. Available from: https://journals.lww.com/10.1097/XEB.0000000000000239
60. Tabak RG, Duggan K, Smith C, Aisaka K, Moreland-Russell S, Brownson RC. Assessing Capacity for Sustainability of Effective Programs and Policies in Local Health Departments. J Public Heal Manag Pract [Internet]. 2016 Mar;22(2):129–37. Available from: https://journals.lww.com/00124784-201603000-00005
61. Tomioka M, Braun KL. Examining Sustainability Factors for Organizations that Adopted Stanford’s Chronic Disease Self-Management Program. Front Public Heal [Internet]. 2015 Apr 27;2(APR):1–8. Available from: http://journal.frontiersin.org/article/10.3389/fpubh.2014.00140/abstract
62. Van Heerden C, Maree C, Janse van Rensburg ES. Strategies to sustain a quality improvement initiative in neonatal resuscitation. African J Prim Heal Care Fam Med [Internet]. 2016 Apr 22;8(2):958. Available from: http://dx.doi.org/10.4102/phcfm.v8i2.958
63. Zakumumpa H, Bennett S, Ssengooba F. Accounting for variations in ART program sustainability outcomes in health facilities in Uganda: a comparative case study analysis. BMC Health Serv Res [Internet]. 2016 Dec 18;16(1):584. Available from: http://dx.doi.org/10.1186/s12913-016-1833-4
64. Zakumumpa H, Kwiringira J, Rujumba J, Ssengooba F. Assessing the level of institutionalization of donor-funded anti-retroviral therapy (ART) programs in health facilities in Uganda: implications for program sustainability. Glob Health Action [Internet]. 2018 Jan 8;11(1):1523302. Available from: https://doi.org/10.1080/16549716.2018.1523302
