# Supplementary material for: Identifying existing approaches used to evaluate the sustainability of evidence-based interventions in healthcare: an integrative review
Source: Syst Rev. 2022 Oct 15;11:221. doi: 10.1186/s13643-022-02093-1 (PMC9569065; doi:10.1186/s13643-022-02093-1)
Supplement: Supplementary file 4 — Additional file 4. Citation list of included studies. A table of the sustainability outcomes reported in all of the included studies (n=64). The table depicts which of the 9 sustainability outcomes were reported in each study. [file 13643_2022_2093_MOESM4_ESM.docx]

|  | Benefits for patients, staff and stakeholders continue | Initiative activities or components of the intervention continue | Maintenance of relationships, partnerships or networks | Maintenance of new procedures, and policies | Attention and awareness of the problem or issue is continued or increased | Replication, roll-out or scale-up of the initiative | Capacity built within staff, stakeholders and communities continues | Adaptation in response to new evidence or contextual influences | Gaining further funds to continue the initiative and maintain improvements |
| --- | --- | --- | --- | --- | --- | --- | --- | --- | --- |
| Aarons, G. A. et al. | ✓ |  | ✓ | ✓ |  |  |  |  | ✓ |
| Abimbola | ✓ |  |  |  |  |  | ✓ |  |  |
| Allchin |  | ✓ | ✓ | ✓ |  |  | ✓ | ✓ |  |
| Ament, S. M. et al. | ✓ | ✓ | ✓ | ✓ |  | ✓ |  | ✓ |  |
| Azeredo, T. B. et al. | ✓ |  |  | ✓ |  |  |  | ✓ | ✓ |
| Baloh |  |  | ✓ |  |  | ✓ |  |  |  |
| Belizan, M. | ✓ | ✓ | ✓ | ✓ | ✓ |  | ✓ |  |  |
| Belostotsky |  |  |  |  |  |  |  |  |  |
| Berendsen, B. A. et al. | ✓ | ✓ |  |  |  |  |  | ✓ |  |
| Blanchet, K., James, P. | ✓ | ✓ |  |  |  |  |  | ✓ |  |
| Bond GR, | ✓ | ✓ | ✓ |  |  | ✓ |  |  | ✓ |
| Bray, P. et al. | ✓ | ✓ | ✓ | ✓ | ✓ |  | ✓ |  | ✓ |
| Bridges et al | ✓ |  |  |  |  |  |  |  |  |
| Butow | ✓ |  |  |  | ✓ | ✓ | ✓ | ✓ |  |
| Campbell, S. et al. | ✓ |  |  |  |  | ✓ |  |  | ✓ |
| Carlfjord, S., Lindberg, M., Andersson, A. |  |  |  |  |  |  |  |  |  |
| Carstensen | ✓ | ✓ | ✓ | ✓ |  |  |  | ✓ |  |
| Curry, S. J., Mermelstein, R. J., Sporer, A. K. | ✓ | ✓ |  |  | ✓ |  | ✓ |  |  |
| Dharmayat et al. | ✓ |  | ✓ |  |  |  |  |  |  |
| Doyle, C. et al. |  |  |  |  |  |  |  |  |  |
| Eakin, M. N. et al. | ✓ | ✓ | ✓ | ✓ | ✓ | ✓ |  |  | ✓ |
| El Bcheraoui, C. et al. | ✓ | ✓ | ✓ | ✓ | ✓ | ✓ | ✓ | ✓ |  |
| Finch, T. L. et al. | ✓ | ✓ | ✓ | ✓ | ✓ | ✓ | ✓ | ✓ |  |
| Flynn |  | ✓ |  |  |  |  |  | ✓ |  |
| Ford JH II, Krahn D, Wise M, Oliver KA. | ✓ | ✓ |  | ✓ |  |  | ✓ |  |  |
| Ford, J. H., 2nd et al. | ✓ | ✓ |  |  | ✓ |  |  |  | ✓ |
| Frykman, M. et al. |  |  |  |  |  |  |  |  |  |
| Garst, J. et al. | ✓ |  | ✓ | ✓ | ✓ | ✓ | ✓ | ✓ |  |
| Graham | ✓ | ✓ | ✓ | ✓ | ✓ |  |  |  |  |
| Greenhalgh, T. et al. | ✓ | ✓ | ✓ | ✓ |  | ✓ | ✓ | ✓ |  |
| Grow HMG, | ✓ | ✓ | ✓ | ✓ |  | ✓ | ✓ | ✓ |  |
| Healey | ✓ | ✓ | ✓ | ✓ | ✓ | ✓ | ✓ |  | ✓ |
| Hovlid, E. et al. | ✓ | ✓ |  |  |  |  | ✓ | ✓ |  |
| Hunter SB, Han B, |  |  |  |  |  |  |  |  |  |
| Jones | ✓ |  | ✓ | ✓ |  |  | ✓ | ✓ |  |
| Kacholi | ✓ |  |  |  |  |  |  |  |  |
| Kastner, M. et al. |  |  |  |  |  |  |  |  |  |
| Kempen | ✓ |  | ✓ | ✓ | ✓ | ✓ | ✓ |  | ✓ |
| Kennedy |  | ✓ | ✓ | ✓ | ✓ | ✓ | ✓ | ✓ |  |
| King, D. K. | ✓ | ✓ | ✓ | ✓ |  | ✓ | ✓ | ✓ |  |
| Klinga, C. et al. |  |  | ✓ | ✓ |  |  | ✓ | ✓ |  |
| Knapp H, Haged | ✓ | ✓ | ✓ | ✓ |  |  | ✓ | ✓ |  |
| Kosse | ✓ |  |  |  |  |  |  |  |  |
| Lillvis |  |  |  |  |  |  |  |  |  |
| Lindholm | ✓ | ✓ |  |  |  |  |  |  |  |
| Mahomed OH, | ✓ | ✓ |  |  |  |  |  |  |  |
| Morden, A. et al. | ✓ | ✓ |  | ✓ |  |  |  |  |  |
| Nazar, H., Nazar, Z. |  |  | ✓ |  |  |  |  |  |  |
| Nordmark, S., Zingmark, K.,Lindberg, I. | ✓ |  |  | ✓ |  |  |  |  |  |
| Palinkas |  |  | ✓ |  |  |  |  |  | ✓ |
| Pomey M-P, | ✓ | ✓ | ✓ | ✓ | ✓ | ✓ | ✓ | ✓ | ✓ |
| Rasschaert F, Decroo T, Remartinez D, Telfer B, Lessitala F, Biot M, et al. | ✓ | ✓ | ✓ |  | ✓ | ✓ | ✓ | ✓ |  |
| Seppey M, Ridde V, Toure L, et al. | ✓ | ✓ |  |  |  |  | ✓ | ✓ |  |
| Smith ML, Durrett NK, Schneider EC, | ✓ |  | ✓ |  |  |  |  |  |  |
| Spassiani |  | ✓ | ✓ |  |  |  |  | ✓ | ✓ |
| Stoll, S. et al. |  |  | ✓ | ✓ | ✓ |  | ✓ | ✓ | ✓ |
| Stolldorf | ✓ |  |  |  |  |  |  | ✓ |  |
| Stolldorf | ✓ | ✓ | ✓ | ✓ |  | ✓ | ✓ | ✓ |  |
| Sving | ✓ | ✓ |  | ✓ | ✓ |  | ✓ | ✓ | ✓ |
| Tabak RG, Duggan K, Smith C, - |  |  |  |  |  |  |  |  |  |
| Tomioka M, Braun KL. | ✓ | ✓ | ✓ | ✓ |  |  | ✓ | ✓ | ✓ |
| Van Heerden, C. Maree, C. Janse van Rensburg, E. S. | ✓ | ✓ | ✓ |  |  |  | ✓ |  |  |
| Zakumumpa, H., Bennett, S., Ssengooba, F. |  | ✓ | ✓ | ✓ | ✓ |  |  |  | ✓ |
| Zakumumpa, H., Kwiringira, J.,Rujumba, J., Ssengooba, F. |  | ✓ |  | ✓ |  |  | ✓ | ✓ |  |
| Total: | ***45*** | ***36*** | ***34*** | ***31*** | ***18*** | ***18*** | ***29*** | ***29*** | ***16*** |
